# Flow Management to Control Excessive Growth of Macrophytes – An Assessment Based on Habitat Suitability Modeling

**DOI:** 10.3389/fpls.2018.00356

**Published:** 2018-03-19

**Authors:** Konstantin Ochs, Rui P. Rivaes, Teresa Ferreira, Gregory Egger

**Affiliations:** ^1^Forest Research Centre, Instituto Superior de Agronomia, University of Lisbon, Lisbon, Portugal; ^2^Department of Wetland Ecology, Institute of Geography and Geoecology, Karlsruhe Institute of Technology, Rastatt, Germany

**Keywords:** aquatic macrophytes, habitat suitability modeling, flow regulation, invasive species, *Myriophyllum aquaticum*, IFIM

## Abstract

Mediterranean rivers in intensive agricultural watersheds usually display outgrowths of macrophytes – notably alien species – due to a combination of high concentrations of nutrients in the water runoff and low flows resulting from water abstraction for irrigation. Standard mechanical and chemical control is used to mitigate the problems associated with excessive growth of plant biomass: mainly less drainage capacity and higher flood risk. However, such control measures are cost and labor-intensive and do not present long-term efficiency. Although the high sensitivity of aquatic vegetation to instream hydraulic conditions is well known, management approaches based on flow management remain relatively unexplored. The aim of our study was therefore to apply physical habitat simulation techniques promoted by the Instream Flow Incremental Method (IFIM) to aquatic macrophytes – the first time it has been applied in this context – in order to model shifts in habitat suitability under different flow scenarios in the Sorraia river in central Portugal. We used this approach to test whether the risk of invasion and channel encroachment by nuisance species can be controlled by setting minimum annual flows. We used 960 randomly distributed survey points to analyze the habitat suitability for the most important aquatic species (including the invasive Brazilian milfoil *Myriophyllum aquaticum*, *Sparganium erectum*, and *Potamogeton crispus*) in regard to the physical parameters ‘flow velocity,’ ‘water depth,’ and ‘substrate size’. We chose the lowest discharge period of the year in order to assess the hydraulic conditions while disturbances were at a low-point, thus allowing aquatic vegetation establishment and subsistence. We then used the two-dimensional hydraulic River2D software to model the potential habitat availability for different flow conditions based on the site-specific habitat suitability index for each physical parameter and species. Our results show that the growth and distribution of macrophytes in the hydrologically stable vegetation period is primarily a function of the local physical instream condition. Using site-specific preference curves and a two-dimensional hydraulic model, it was possible to determine minimum annual flows that might prevent the excessive growth and channel encroachment caused by *Myriophyllum aquaticum*.

## Introduction

Aquatic macrophytes play an important role in riverine ecosystems, providing habitats for many organisms and affecting the hydraulic and chemical instream condition (Carpenter and Lodge, 1986). Their distribution and abundance are primarily determined by the hydrologic regime (frequency, duration, and intensity of flood events) (Riis and Biggs, 2003; Franklin et al., 2008), which controls biomass loss and gain processes. Whereas loss processes are caused by increased drag forces during high flood events that cause stem breakage and uprooting of plants, biomass gain processes happen while disturbances are absent during medium to low flow conditions (Riis et al., 2008). In these stable interflood periods, macrophyte growth is controlled by several physical and chemical factors, including flow velocity and depth (Chambers et al., 1991; Riis and Biggs, 2003), light availability (Carr et al., 1997; Köhler et al., 2010), water temperature (Barko et al., 1986; Carr et al., 1997), and riverbed grain size (Baattrup-Pedersen and Riis, 1999), as well as the nutrient content of the riverbed and water (Barko et al., 1986; Demars and Edwards, 2009). Anthropogenic disturbances, such as high nutrient concentrations from water runoff (Jones et al., 2002; Mainstone and Parr, 2002), low suspended sediment concentrations and the resulting increase in light availability from river damming (Madsen et al., 2001; Köhler et al., 2010) and stabilization of the flow regime (less floods) (Riis and Biggs, 2003; Franklin et al., 2008) can alter the ecological equilibrium of the system and have been shown to stimulate excessive growth of aquatic vegetation, notably invasive alien species (Bunn and Arthington, 2002). This is known to cause various forms of ecological and economic damage (Brundu, 2014), including changes in species composition and richness (Bunn and Arthington, 2002; O’Hare et al., 2006), increased flood risk through higher flow resistance (Vereecken et al., 2006; Nikora et al., 2008), and interferences with human water uses such as water abstraction, hydropower, recreation and river navigation (Halstead et al., 2003; Gómez et al., 2013). Management of aquatic macrophytes by mechanical (cutting) or chemical (herbicides) means is therefore common practice in many rivers worldwide (Madsen, 2000; Hussner et al., 2017).

Especially in regulated Mediterranean rivers flowing through intensive agricultural watersheds and presenting prolonged spells of low flows the outgrowth of aquatic vegetation, and notably alien species, is a common phenomenon (Ferreira and Moreira, 1999; Aguiar and Ferreira, 2013). Despite their high costs, mechanical control measures are widely applied in Portugal (Moreira et al., 1999).

Although the growth and distribution of aquatic macrophytes in unshaded streams is mainly influenced by local hydraulic conditions (depth/velocity/sediments) (Chambers et al., 1991; Riis and Biggs, 2003), whose impact overshadows that of hydrochemistry (Steffen et al., 2014), little attention has thus far been paid to the possibility that channel encroachment and invasion can be controlled by establishing minimum annual flows. One common way of exploring the effectiveness of such ecosystem-regulation measures is ecological modeling, because model-based testing is faster and requires less financial inputs than actual physical experiments (Perona et al., 2009; Schmolke et al., 2010). Modeling species distribution or habitat suitability as functions of environmental factors is frequently used to provide spatial decision support for environmental management, weed or pest species risk assessments and studies of climate-change impacts (Franklin, 2013). In the case of river ecosystems, the instream flow incremental method (IFIM) (Bovee, 1982; Raleigh et al., 1986) is probably still the most widely used and accepted methodology for predicting the response of aquatic biota to the instream physical condition (Jowett et al., 2008; Conallin et al., 2010). However, its concepts have never been directly applied to the management of aquatic macrophytes.

Against this background, the main aim of this study was, for the first time, to apply and validate the hydraulic habitat modeling techniques promoted by the IFIM for the assessment of annual minimum flows with the ability to reduce the risk of channel encroachment and invasion by the alien *Myriophyllum aquaticum* in a heavily regulated Mediterranean river. Our hypothesis was that summer low flows further intensified by water abstraction for irrigation create physical instream conditions that favor the excessive growth of *M. aquaticum* over the autochthonous *Sparganium erectum* and *Potamogeton crispus*, and that this situation can be mitigated by establishing minimum flows above a certain threshold.

## Methodology

### Study Area

**FIGURE 1:**
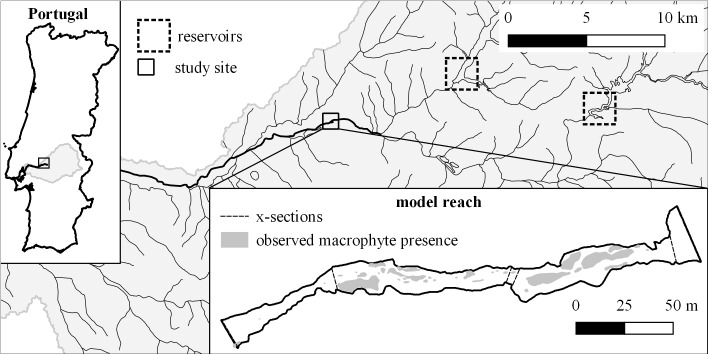
Location of the study site in Portugal and the Sorraia basin (rectangle), the position of the two largest reservoirs (dotted rectangles) and the wetted area of the model reach at *Q* = 0.3 m^3^/s, the location of the *x*-sections used for the hydraulic model calibration (including boundaries), and the observed macrophyte presence used to validate the habitat suitability model.

The study area is located along the Sorraia river in central Portugal (**Figure 1**). The river basin has an accumulated area of 7719 km^2^ and a semi-arid Mediterranean climate in which most of the annual rainfall (600–800 mm) occurs between October and May and the mean annual temperature is 16–19°C. The fieldwork was carried out along a naturally braided, unconfined segment of the river. The riparian corridor from the edge of the active channel to the adjacent agricultural areas consists mostly of willow shrubs, and willows (*Salix alba*) in higher areas, and extends an average of 60 m either side of the river. The active channel has an average width of 15 m and is mostly unshaded. The segment’s substrate is dominated by sands, gravels and cobbles. Surrounding land is given over to intensive rice, maize, and tomato cultivation. We chose a calibration reach of approximately 1000 m in length for the model-building, and a model reach with a length of 320 m directly downstream for testing and application. Both reaches contain all the different mesohabitats (pool/run/riffle) found in the segment.

**FIGURE 2:**
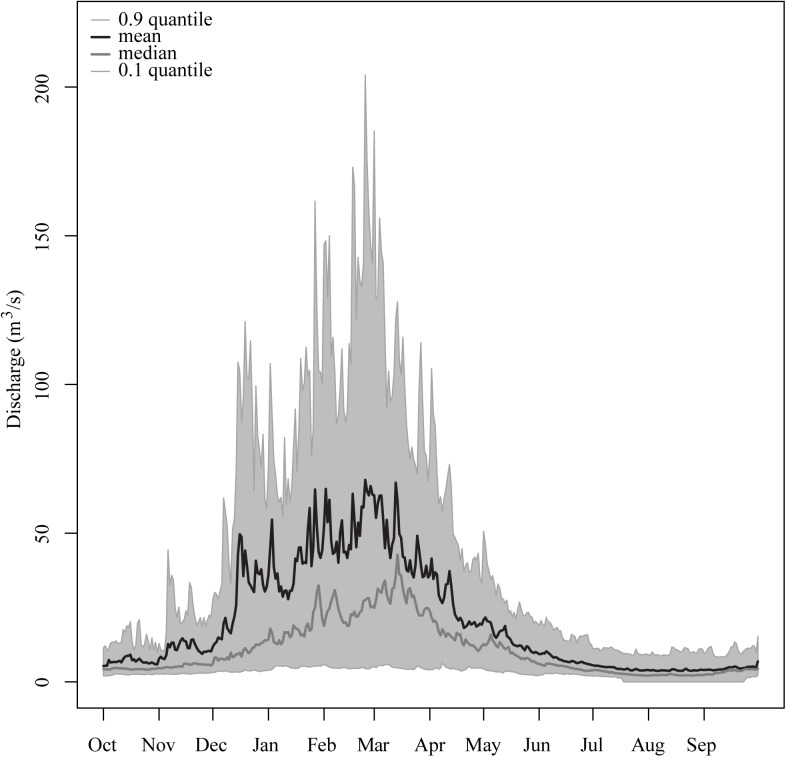
Summary of the flow regime of the Sorraia river (available data for 1933–1980 from the “Ponte Coruche” Gauging station): The area between the upper (0.9) and lower (0.1) quantiles is shaded gray; the black line represents the mean daily discharge; the gray line represents the median daily discharge.

The Sorraia’s hydrological regime presents a high intra- and inter-annual discharge variability, which is characteristic of Mediterranean watersheds (Gasith and Resh, 1999). The mean annual discharge is 20.14 m^3^/s (available data for 1933–1980, “Ponte Coruche” Gauging station). The heaviest winter floods can attain 887 m^3^/s, while during the summer months (June–September) the mean discharge is 3.2 m^3^/s and low flow spells are common (**Figure 2**).

The flow regime is heavily regulated by a system of reservoirs, weirs and canals that was implemented between 1933 and 1958. Water abstraction for agricultural irrigation is managed by a local farmers’ association, which mechanically cleans the river channel of aquatic macrophytes and riparian vegetation every few years to reduce flood risk.

### Aquatic Vegetation

The main aquatic macrophyte species occurring in study area are *M. aquaticum*, *S. erectum*, and *P. crispus*. Other species that presented less prevalence and were therefore not considered were *Ceratophyllum demersum* and *Typha domingensis.* Based on their growth form, *M. aquaticum* and *S. erectum* are classified as sediment-rooted plants with floating or emergent shoots/leaves, whereas *P. crispus* is a sediment-rooted submerged plant (Den Hartog and Van Der Velde, 1988). Following the definition of Pyšek et al. (2004) *M. aquaticum* is considered an invasive species in Portugal. It was first reported in 1936 (Aguiar and Ferreira, 2013), but massive spreading was only observed in the 1970s (Moreira et al., 1999). *M. aquaticum* is displacing native aquatic species, including *P. crispus* and *C. demersum*, in many parts of the River Tagus (Ferreira and Moreira, 1995).

### IFIM Overview

The instream flow incremental methodology (Bovee, 1982; Raleigh et al., 1986) is a framework which the United States Fish and Wildlife Service developed in the late 1970s to determine appropriate minimum annual flows by considering the effects of flow changes on instream habitat suitability of aquatic biota. It is probably still the most widely used and accepted methodology for predicting the response of aquatic biota to the instream physical condition (Jowett et al., 2008; Conallin et al., 2010). Its main feature is a hydraulic habitat suitability model that can be separated into a hydraulic component and a habitat component. The hydraulic model predicts water velocity, depth and other hydraulic variables. The habitat model is based on local habitat suitability curves (HSC) that describe the optimum range of a physical parameter affecting the species and are built on expert knowledge or field analyses of local species occurrence and habitat availability. Integrating the two components makes it possible to calculate a composite suitability index (CSI) that combines the suitability information for each physical parameter at a given flow. The weighted usable area (WUA) for the target species is quantified by multiplying the CSI by its area of influence. In order to assess an appropriate minimum annual flow, the hydraulic habitat suitability model is applied to a range of flows to produce a WUA-vs.-discharge graph.

### Hydraulic Habitat Suitability Modeling

In order to calibrate (train) the habitat suitability model, a total of 961 sample points were distributed systematically (2 m × 2 m), with a randomly chosen starting point along each mesohabitat (pool, run, and riffle) found in the calibration reach. The mesohabitats were visually delimited in the field.

The occurrence of the main macrophyte species and physical habitat characteristics – flow velocity, water depth and grain size of the bed material – were analyzed at each sample point. The fieldwork was done in August 2016 and July 2017, during measured discharges of around 0.3 m^3^/s. We chose the lowest discharge period of the year in order to assess the hydraulic conditions during the period of least disturbance, which allows aquatic vegetation establishment and subsistence. Locations shaded by riparian vegetation (less than 5% of the analyzed reach) were excluded, since in this situation aquatic plant growth is mainly constrained by insufficient light (Carr et al., 1997). Depths were measured with a simple meter ruler and classified in intervals of 20 cm. Flow velocities were measured with a water flow probe (model FP101, Global Water Instrumentation, United States) positioned in the flow direction at 60% of the flow depth and using 0.05 m/s intervals. The bed grain size was assessed visually and classified according to the Wentworth scale (sand: 0.62–2 mm; gravel: 2–64 mm; cobble: 64–256 mm). The habitat preferences for *M. aquaticum*, *S. erectum*, and *P. crispus* were then calculated by dividing habitat-utilization (amount of species occurrences in each class of the physical parameters) by habitat-availability (total amount of each class of the physical parameters). The final preference values were normalized, from a minimum value of 0 for unsuitable to 1.0 for optimal habitats (the class of the physical parameter with the highest amount of species occurrences), and expressed as a HSC for each physical parameter.

In order to apply and test the hydraulic habitat suitability model, we selected a 320 m-long reach directly downstream from the calibration reach. We chose a two-dimensional approach for the hydraulic simulation: the River2D model (Steffler and Blackburn, 2002). Two-dimensional hydraulic models predict depth and velocity laterally and longitudinally along the whole length of the river channel. They are therefore better able to simulate the complex flow patterns found in braided rivers than the more conventional (with regard to the IFIM) one-dimensional models that only predict depth and velocity across channel transects (Benjankar et al., 2015). The topography of the riverbed of the model reach, which is the main input into the hydraulic model, was measured in July 2016 with a Leica TCR703 Total Station (angle accuracy 3″) along 970 points. The initial bed roughness values were estimated based on substrate size and vegetation distribution. To determine the boundary condition and calibrate the model, water depth and velocity were assessed along six transects including the down- and upstream cross-section, with measurements taken every 20 cm along the cross-section. The hydraulic model was calibrated by adjusting bed roughness until simulated water surface elevations matched measured water surface elevations.

The model was then used to simulate the physical instream conditions for a series of potential annual minimum flows of between 0.3 and 10 m^3^/s, representing a common flow range during the vegetation period. The WUA concept was used to evaluate the shift in habitat suitability for each discharge (Bovee, 1982). The WUA computation is based on the habitat suitability evaluated at every node of the topographic mesh and the “tributary area” of that node. We also calculated the Hydraulic Habit Suitability (HHS) for each discharge by dividing the WUA by the inundated area. The HHS can be understood as the percentage of the WUA from the inundated area at a given discharge. A value of 1 would mean that the whole of the wetted area classifies as usable area for a certain species or species group.

We used two different methods to calculate the habitat suitability. The classical, deterministic approach of the IFIM calculates a CSI as the geometric mean of the separate suitability indices for depth, velocity, and substrate size. It is directly integrated into the River2D Model on the basis of the HSC for each species.

$$CSI=\sqrt[{3}]{\left(VSI\times DSI\times SSI\right)}$$

VSI – Velocity Suitability Index
DSI – Depth Suitability Index
SSI – Substrate Suitability Index

In addition to the deterministic approach, we computed the habitat suitability for each species based on the random forest algorithm (RF) for classification (Breiman, 2001). We used the R package “randomForest” (Liaw and Wiener, 2002) to grow 1000 trees based on bootstrap samples of the same training data as that used to build the HSC, and incorporated 50% class weights into the classifier to account for the low prevalence of *P. crispus* and *S. erectum*.

### Model Validation

We mapped the true presence and absence of the main macrophyte species (*M. aquaticum*, *S. erectum*, and *P. crispus*) in the model reach with a Global Positioning System unit (Ashtech, model Mobile Mapper 100; accuracy < 50 cm) during the same period (July/August) and with the same discharge (0.3 m^3^/s) as those when the data for the model calibration was collected. We then modeled the macrophyte distribution using the deterministic and the random forest approach based on the hydraulic simulation for the same discharge, and tested the agreement between observed and predicted distribution by assessing the area under the receiver operating characteristic curve (AUC) (Fielding and Bell, 1997). The AUC of a model is equivalent to the probability that the model will rank a randomly chosen species-presence site higher than a randomly chosen absence site. In addition, we transformed the predicted occurrence probabilities of both models to a binary presence/absence format for each species using the threshold of occurrence that maximizes the sum of sensitivity and specificity (Cantor et al., 1999; Liu et al., 2005). In order to assess the accuracy of the binary classification, we used the “True Skill Statistic” (TSS; sensitivity + specificity – 1), because it accounts for the effect of the species prevalence (Allouche et al., 2006). All accuracy measurements were carried out using the R package “SDMtools” (VanDerWal et al., 2014).

In order to investigate whether our models accounted for all the factors causing the species’ distributional pattern, we checked the observed species occurrence in the model reach for spatial autocorrelation using the Ripley’s K function, and tested the error between observed and predicted species occurrence for clustering with the Moran’s I index. The spatial analyses were done with the spatial statistics toolbox from ArcGIS for desktop (version 10.4.1).

## Results

### Habitat Suitability Curves

The habitat sampling resulted in 224 *M. aquaticum*, 135 *P. crispus*, and 85 *S. erectum* presences in a total of 961 habitat samples.

**FIGURE 3:**
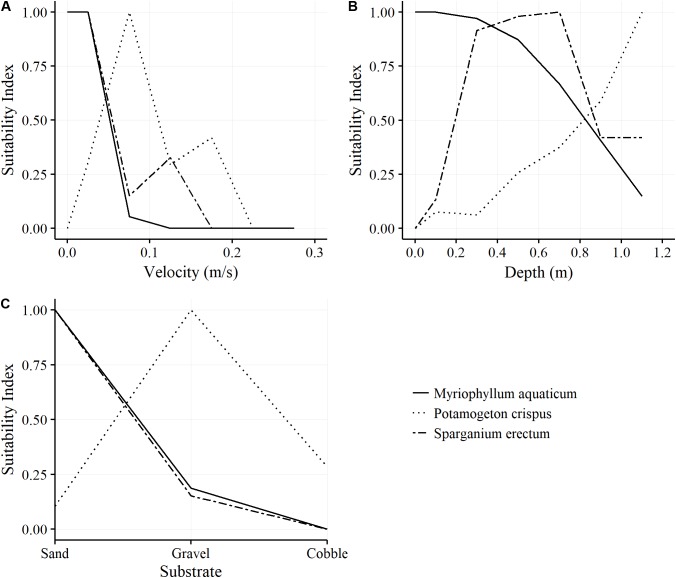
Suitability Index (SI) with regard to flow velocity **(A)**, water depth **(B)**, and substrate size of the bed material **(C)** for *Myriophyllum aquaticum*, *Potamogeton crispus*, and *Sparganium erectum;* values of 1 signify optimal and values of 0 signify no suitability.

*Myriophyllum aquaticum* displayed a substantial liking for low flow conditions, only having colonized areas with relatively slow velocities and low depth. It was already nearly absent at velocities over 0.1 m/s. The most suitable depths were 0–20 cm. In addition, it was found almost exclusively on sandy substrate. On the contrary, *P. crispus* seemed to prefer higher-flow areas. Its greatest presence occurred in medium velocities of 0.08–0.2 m/s and it clearly favored depths of more than 80 cm. Its preferred substrate was gravel. *S. erectum* displayed a preference profile similar to that of *M. aquaticum*, but was more tolerant of greater depth. The results show a distinct preference profile of the exotic *M. aquaticum* with regard to flow velocity and water depth (**Figure 3**).

### Model Validation

In overall terms, the hydraulic habitat model based on the deterministic approach displayed a good discriminatory ability. In the case of *M. aquaticum*, accuracy was even in the excellent range (AUC = 0.9), while for *P. crispus* it was good (AUC = 0.87), and for *S. erectum* fair (AUC = 0.79). The performance of the binary classification differed more drastically between the species. Considering a threshold of occurrence for *M. aquaticum* of 0.24, the TSS score of the model was 0.66. It correctly predicted 86% of the actual presences (sensitivity) and 80% of the actual absences (specificity). The occurrence threshold for *Potamogeton* was set to 0.24. The TSS score was 0.62. Its occurrence was correctly predicted in 88% of cases, and its absence in 70%. The model’s worst performance was for *S. erectum*, with an occurrence threshold of 0.08 (TSS = 0.44; Sensitivity = 0.7; Specificity = 0.66).

The random forest model did not perform as well as the deterministic approach. On the contrary, only the prediction of *M. aquaticum* achieved a similar accuracy (AUC = 0.85), whereas the predictions for *P. crispus* (AUC = 0.7) and *S. erectum* (AUC = 0.65) were less accurate. This was also visible in the binary prediction. Considering a threshold of occurrence of 0.6 for *M. aquaticum*, the model’s TSS score was 0.66 (sensitivity = 0.8; specificity = 0.86). The prediction of *Potamogeton* based on a threshold of 0.5 returned a TSS score of 0.38 (sensitivity = 0.66; specificity = 0.72). Once again, the model performed worst for *S. erectum* (threshold = 0.2; TSS = 0.28; sensitivity = 0.66; specificity = 0.62).

The species occurrence as well as the errors between the observed and predicted distributions presented a similar degree of positive spatial autocorrelation (clustered pattern), indicating that although our models have a medium to high degree of accuracy, they do not account for all the factors explaining the species distribution.

### Weighted Usable Area and Hydraulic Habitat Suitability

We only used the deterministic modeling approach to analyze the shifts in habitat suitability for incremental flows because of its better predictive performance.

**FIGURE 4:**
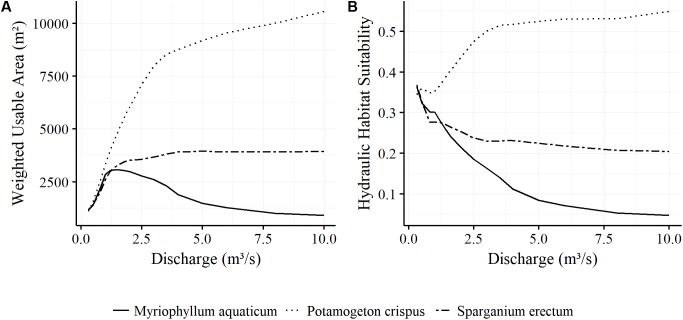
Weighted Usable Area **(A)** and Hydraulic Habitat Suitability **(B)** of the main species found in the study area as a function of discharge.

The preference of *M. aquaticum* for low flow conditions is also reflected in the development of the WUA. From 1167 m^2^ at *Q* = 0.3 m^3^/s, it rapidly increases until it reaches its maximum of 3085 m^2^ at *Q* = 1.4 m^3^/s. The WUA drops steadily after that, although the inundated and therefore potentially invadable area continues to increase with rising flows. The WUA decreases more slowly from *Q* = 5 m^3^/s to *Q* = 8 m^3^/s, after which it remains nearly constant. At *Q* = 0.3 m^3^/s *P. crispus* has a WUA of 1017 m^2^, slightly lower than that of *M. aquaticum* and *S. erectum*. However, this then sharply increases, so that at *Q* = 3 m^3^/s the *P. crispus* WUA of 8004 m^2^ is already three times higher than that of *M. aquaticum*. After that, the upward trend continues more slowly, but steadily. At *Q* = 10 m^3^/s, the *P. crispus* WUA of 10569 m^2^ is over 10 times that of the invaders. The development of the WUA of *S. erectum* initially appears to be similar to that of *M. aquaticum*. However, it continues to gain area until *Q* = 3.5 m^3^/s, after which the WUA stays relatively constant at around 3900 m^2^, whereas the *M. aquaticum* WUA experiences a steady decline over the same range (**Figure 4A**).

In the case of *M. aquaticum*, the HHS trends continuously downward as discharge increases. Whereas 36% of the wetted area is potentially suitable at *Q* = 0.3 m^3^/s, only about 10% remains suitable at *Q* = 4 m^3^/s. *P. crispus* experiences an increase in HHS with rising flows. The HHS only decreases slightly at around *Q* = 1 m^3^/s, due to a large increase in wetted area. From *Q* = 3.5 m^3^/s onward, the rate of change in HHS decreases. *S. erectum* also experiences a decline in HHS, sharply at first, to levels below even those of *M. aquaticum*, but remains nearly constant from *Q* = 2.5 m^3^/s onward (**Figure 4B**).

## Discussion

In this study we wanted to explore setting minimum annual flows as an alternative management approach for controlling excessive growth of macrophytes and invasion by *M. aquaticum* during the vegetation period in the Sorraia river. Following IFIM principles, we built a hydraulic habitat suitability model for *M. aquaticum*, *S. erectum*, and *P. crispus*, applied it to a range of discharges, and analyzed the changes in WUA and HHS. Our hypothesis was that low summer flows intensified by water abstraction for irrigation create physical instream conditions that stimulate excessive growth of *M. aquaticum*, and that this situation can be mitigated by establishing minimum flows above a certain threshold.

The modeling results support our hypothesis that the growth and distribution of macrophytes in interflood periods is primarily a function of the local physical instream condition, which is especially favorable to an invasion of *M. aquaticum* during the low flow range. Habitat suitable for *M. aquaticum* already declines above flows of 1.4 m^3^/s, while the autochthonous species, and especially *P. crispus*, continue to gain ground. It would therefore seem possible to reduce the risk of invasion and favor a more natural species composition by setting annual minimum flows. The combination of the artificial approximation of the habitat availability for both the exotic and the autochthonous species caused by stable periods of flows under 1.4 m^3^/s and the greater competitive ability of *M. aquaticum* may be the reason for the latter’s successful expansion. Given that the mean annual flow during the vegetation period is 3.2 m^3^/s, it may well be that water managers can establish minimum annual flows above the 1.4 m^3^/s threshold and thereby avert this situation. This is an important result that can improve river restoration projects by preventing the degradation of natural aquatic vegetation communities.

However, we also observed that for the low flow range (0.3–1.4 m^3^/s), the WUA actually increases for *M. aquaticum* and that the rate of change in habitat suitability for all species is lower with high flows than with low flows. The explanation for this is that the suitable areas are concentrated in shallow waters along the banks of the stream, and these shallow areas initially increase when the river enters the floodplain and then remain relatively constant in size. In the case of *M. aquaticum*, this means that the WUA remains relatively constant above a discharge of 7 m^3^/s. Setting minimum annual flows will therefore not completely prevent an invasion; but it can contribute to an environmental flow regime that privileges autochthonous aquatic species and strengthens their competitive performance.

One major criticism of the IFIM habitat simulation to keep in mind when interpreting the results is the usage of the term WUA (Mathur et al., 1985), because it suggests a spatial extension of usable habitat when in fact it only actually describes the overall probability of use. So when we assess the effects of flow changes on aquatic biota, it is the shape of the WUA response curve that is more important than the magnitude (Jowett et al., 2008).

In addition, as with all modeling approaches, there are a number of different uncertainties that should be considered when interpreting the results.

### Environmental Factors

Our study is based on the assumption that in hydrologically stable periods, physical habitat characteristics are the main limiting factor for aquatic species in streams. Indeed, several studies argue that flow velocity is the main environmental factor controlling the abundance and distribution of aquatic macrophytes (Chambers et al., 1991; Baattrup-Pedersen and Riis, 1999; Madsen et al., 2001; Janauer et al., 2010). Most studies relate the limiting effect of higher flow velocities on plant growth to increased drag forces on the plants and their anchoring ground, causing uprooting, or less frequently, stem breakage (Chambers et al., 1991; Riis and Biggs, 2003). However, a more recent study (Pollen-Bankhead et al., 2011) indicates that the preference of macrophytes for low velocities is less related to the drag forces on the plants and more to the conditions controlling erosion and deposition of fine substrate materials. The effect of substrate size has mainly been studied with regard to the distribution patterns of macrophytes, and not in terms of changes in biomass (Baattrup-Pedersen and Riis, 1999; Riis and Biggs, 2001; O’Hare et al., 2006). The findings indicate a niche separation between macrophytes based on different substrate size preferences. Apparently, submerged species favor coarser substrates (gravel and boulder), whereas species that grow both submerged and emergent, and species that only grow emergent, were associated with finer substrates (sand) typical of low flow conditions. This is coherent with our results. The influence of flow depth has been related to light availability, which decreases with greater depth (Koch, 2001). In situations of high turbidity or direct shading, for example through overhanging vegetation, light availability can also become the main limiting factor, which is why we excluded sample sites with these characteristics (Köhler et al., 2010). Temperature is also known to influence the growth rate of aquatic plants (Koch, 2001). It can, however, be assumed that temperature alterations in the analyzed flow range are marginal and are indirectly covered by the effects of velocity and depth (Gu et al., 1998). Besides the physical factors, geochemical properties of the stream and especially nutrient availability are known to have an influence on aquatic biota (Koch, 2001). Unnatural high concentrations of phosphorus, as often occur in agricultural watersheds, can stimulate excessive macrophyte growth (Mainstone and Parr, 2002). However, these factors are still most probably overshadowed by the hydraulic conditions (Barendregt and Bio, 2003; Steffen et al., 2014), as is also indicated by the high accuracy of our model.

### Data Collection/Model Calibration

Different forms of data analysis for generating the HSC for each environmental factor are distinguished for the IFIM (Bovee, 1986): (a) expert knowledge; (b) analyses of actual habitat conditions used by the species (or presence only data); and (c) *in situ* species occurrence and habitat availability data (or presence/absence data). We based our model calibration solely on actual presence/absence data (c). It is the most highly recommended of the three methods (Jowett et al., 2008), and the only one that permits an estimation of the true probability of observing a species at a site (Guillera-Arroita et al., 2015). We kept geographical sampling bias to a minimum by selecting a calibration (training) reach and a model reach from the same river segment, and by applying a stratified, systematic sampling design with a random starting point. The detection error, which is crucial to the performance of many habitat suitability models (Lahoz-Monfort et al., 2014), can be considered negligible because of the sampling design, the small number of different species and their sessility.

Model calibration errors can also affect the two-dimensional hydraulic modeling, which can be compromised due to the collection of insufficient or erroneous bed topography data, insufficiently detailed substrate distribution mapping, erroneous model calibration, or failure to include effects of the bed topography upstream of the study site in the model (Jowett and Duncan, 2012).

### Model Algorithm

The IFIM commonly uses a univariate algorithm to relate the abiotic characteristics to actual habitat suitability (Conallin et al., 2010). The univariate derivation of the CSI is criticized for being based on the assumption that organisms select each habitat variable independently, ignoring interactions and cumulative effects between them (Ahmadi-Nedushan et al., 2006), such as the influence of velocity on substrate stability and composition (Shields, 1936). Multivariate statistical models, such as Generalized Additive Models (Milner et al., 2001) and Artificial Neural Networks (Gozlan et al., 1999), are alternative means of fitting the suitability data that are able to account for interactions between the variables and overcome the problem of independence (Ahmadi-Nedushan et al., 2006). Another, increasingly popular, approach is the use of “fuzzy logic” to define a set of rules that classifies suitability according to a combination of different environmental factors. It allows consideration of uncertain measurements and vague expert knowledge, as well as multivariate effects, without requiring the input parameters to be independent (Noack et al., 2013). With random forests we also applied a distribution modeling technique that is capable of modeling complex interactions among predictor variables and is considered to have one of the greatest discriminatory capacities (Elith et al., 2006; Cutler et al., 2007).

However, random forest and all other approaches are static and ignore more complex processes that are known to shape the distribution patterns of macrophytes, such as interspecific competition and feedbacks between the plants and the physical environment known as niche construction (Corenblit et al., 2009). The latter has become very evident in the complex relationship between macrophytes and fine sediment, where macrophytes have been observed to create positive growth conditions through retention and stabilization of fine sediments, thereby also interacting with geomorphological processes (Schoelynck et al., 2012).

### Model Validation

Ecological modeling is of little value if the prediction is not tested against independent data (Olden et al., 2002). We therefore separated the study reach from the calibration reach and collected field data in two different years. The overall model prediction capacity at *Q* = 0.3 m^3^/s was assessed as good using the threshold-independent AUC statistic. The binary prediction, and especially the rate of observed absences of the species that fall in pixels of predicted presences (the commission error rate, which equals 1 minus specificity), was less convincing, but can in part be explained by the low prevalence of the species. A distinction must be made between two different types of commission error: real commission errors, in which combinations of environmental conditions that are not within the species’ niche are falsely interpreted as suitable; and apparent commission errors, where absence represents a real feature of the species’ distributional ecology due to interspecific interactions and historical factors (Peterson, 1999). A high commission error is therefore common among species that show a low prevalence, and can be an indicator that the species has not yet conquered the whole of its potential niche. If this interpretation is correct, it would support the use of our model as a screening tool for identifying areas that are at higher risk of invasion.

We can only speculate about the causes of the spatial autocorrelation in the errors between observed and predicted species distribution: disregard of interactions between the predictor variables, omission of important predictors (temperature and nutrients), or ecological processes (dispersal, competition, and niche construction) (Guisan and Thuiller, 2005). However, the model’s good predictive performance against independent data nonetheless proves the usefulness of the IFIM approach for predicting macrophyte distribution.

### Other Management Options and Conclusion

Mechanical methods are the most widely used measures for controlling aquatic macrophytes in both Portugal (Moreira et al., 1999) and Europe as a whole (Hussner et al., 2017). They allow for containment or eradication, depending on the specific technique and frequency of application (Madsen, 2000). Although often regarded as environmentally less harmful, the most common and effective measures like mowing are not species-specific and can both harm non-target aquatic biota and cause sediment resuspension (e.g., Habib and Yousuf, 2014). Worldwide, chemical control is also applied. While proven very effective, even for eradicating nuisance weeds (Champion and Wells, 2014), herbicides will physiologically affect similar native aquatic plants and potentially also indirectly harm fish and invertebrates (Getsinger, 1998). The use of herbicides to control aquatic nuisance weeds is therefore severely restricted in various countries (especially in the EU). Biological measures also present a risk of off-target impacts, both directly and indirectly through alteration of the food web. Physical management methods are distinguished from mechanical techniques, because instead of the plants directly, it is their environment that is manipulated. Several physical techniques can be distinguished: dredging, drawdown, benthic barriers, shading or light attenuation, and nutrient inactivation (Madsen, 2000; Wersal et al., 2013). The control of nuisance weeds through flow regulation fits into the latter category, but has so far received little attention. Flushing flows have been successfully used to eradicate weeds in the Ebro river (Tena et al., 2013). However, frequency and magnitude of discharges (in the range of a 2-year flood) are not a viable option for intensive agricultural watersheds like the Sorraia, where both the side effects of the floodings and the competing water uses have to be considered.

Although most management techniques have some negative side effects on the ecosystem, so do the invasion and extreme growth of alien species. Maintaining minimum discharges in order to prevent channel encroachment may be an ecologically and financially advantageous addition to the range of commonly practiced control measures. We tested this approach by applying habitat suitability modeling techniques that are widely used to evaluate environmental flows and restoration measures aimed at fishes and invertebrates. Based on the specific habitat preferences of *M. aquaticum*, it seems possible to set minimum flows that reduce the invader’s habitat while simultaneously promoting that of autochthonous and less invasive aquatic species. This measure can be recommended with a high level of confidence, given that when the model was checked against independent data, it displayed a good level of accuracy in predicting species distribution.

## Author Contributions

KO, RR, TF, and GE made substantial contributions to the conception and design of the study and the acquisition, analysis, and interpretation of data. They participated in drafting the article and revising it critically for important intellectual content. Gave final approval for the version of the manuscript to be submitted. Took public responsibility for the content.

## Conflict of Interest Statement

The authors declare that the research was conducted in the absence of any commercial or financial relationships that could be construed as a potential conflict of interest.

## References

[B1] Aguiar F. C. Ferreira M. T. (2013). A review Plant invasions in the rivers of the Iberian Peninsula, south-western. *Plant Biosyst.* 147 1107–1119. 10.1080/11263504.2013.861539

[B2] Ahmadi-Nedushan B. St-Hilaire A. Bérubé M. Robichaud É Thiémonge N. Bobée B. (2006). A review of statistical methods for the evaluation of aquatic habitat suitability for instream flow assessment. *River Res. Appl.* 22 503–523. 10.1002/rra.918

[B3] Allouche O. Tsoar A. Kadmon R. (2006). Assessing the accuracy of species distribution models: prevalence, kappa and the true skill statistic (TSS). *J. Appl. Ecol.* 43 1223–1232. 10.1111/j.1365-2664.2006.01214.x

[B4] Baattrup-Pedersen A. Riis T. (1999). Macrophyte diversity and composition in relation to substratum characteristics in regulated and unregulated Danish streams. *Freshw. Biol.* 42 375–385. 10.1046/j.1365-2427.1999.444487.x

[B5] Barendregt A. Bio A. M. F. (2003). Relevant variables to predict macrophyte communities in running waters. *Ecol. Model.* 160 205–217. 10.1016/S0304-3800(02)00254-5

[B6] Barko J. W. Adams M. S. Clesceri N. L. (1986). Environmental factors and their consideration in the management of submersed aquatic vegetation: a review. *J. Aquat. Plant Manage.* 24 1–10.

[B7] Benjankar R. Tonina D. Mckean J. (2015). One-dimensional and two-dimensional hydrodynamic modeling derived flow properties: impacts on aquatic habitat quality predictions. *Earth Surf. Process. Landforms* 40 340–356. 10.1002/esp.3637

[B8] Bovee K. D. (1982). *A Guide to Stream Habitat Analysis Using the Instream Incremental Flow Methodology. Instream Flow Inf. Pap. No. 12 FWS/OBS-82/26*, United States Fish Wildlife Service, Washington, DC.

[B9] Bovee K. D. (1986). *Development and Evaluation of Habitat Suitability Criteria for Use in the Instream Flow Incremental Methodology.* Washingdon, DC: US Fish and Wildlife Service.

[B10] Breiman L. (2001). Random forests. *Mach. Learn.* 45 5–32. 10.1023/A:1010933404324

[B11] Brundu G. (2014). Plant invaders in European and Mediterranean inland waters: profiles, distribution, and threats. *Hydrobiologia* 746 61–79. 10.1007/s10750-014-1910-9

[B12] Bunn S. E. Arthington A. H. (2002). Basic principles and ecological consequences of altered flow regimes for aquatic biodiversity. *Environ. Manage.* 30 492–507. 10.1007/s00267-002-2737-0 12481916

[B13] Cantor S. B. Sun C. C. Tortolero-Luna G. Richards-Kortum R. Follen M. (1999). A comparison of C/B ratios from studies using receiver operating characteristic curve analysis. *J. Clin. Epidemiol.* 52 885–892. 10.1016/S0895-4356(99)00075-X 10529029

[B14] Carpenter S. R. Lodge D. M. (1986). Effects of submersed macrophytes on ecosystem processes. *Aquat. Bot.* 26 341–370. 10.1016/0304-3770(86)90031-8

[B15] Carr G. M. Duthie H. C. Taylor W. D. (1997). Models of aquatic plant productivity: a review of the factors that influence growth. *Aquat. Bot.* 59 195–215. 10.1016/S0304-3770(97)00071-5 21327536

[B16] Chambers P. A. Prepas E. E. Hamilton H. R. Bothwell M. L. (1991). Current velocity and its effect on aquatic macrophytes in flowing waters. *Ecol. Appl.* 1 249–257. 10.2307/1941754 27755769

[B17] Champion P. D. Wells R. D. S. (2014). Proactive management of aquatic weeds to protect the nationally important Northland dune lakes in *Proceedings of 19th Australasian Weeds Conference, Hobart*, 139–142.

[B18] Conallin J. Boegh E. Jensen J. K. (2010). Instream physical habitat modelling types: an analysis as stream hydromorphological modelling tools for EU water resource managers. *Int. J. River Basin Manage.* 8 93–107. 10.1080/15715121003715123

[B19] Corenblit D. Steiger J. Gurnell A. M. Naiman R. J. (2009). Plants intertwine fluvial landform dynamics with ecological succession and natural selection: a niche construction perspective for riparian systems. *Glob. Ecol. Biogeogr.* 18 507–520. 10.1111/j.1466-8238.2009.00461.x

[B20] Cutler D. R. Edwards T. C. Beard K. H. Cutler A. Hess K. T. Gibson J. (2007). Random forests for classification in ecology. *Ecology* 88 2783–2792. 10.1890/07-0539.1 18051647

[B21] Demars B. O. L. Edwards A. C. (2009). Distribution of aquatic macrophytes in contrasting river systems: a critique of compositional-based assessment of water quality. *Sci. Total Environ.* 407 975–990. 10.1016/j.scitotenv.2008.09.012 18977514

[B22] Den Hartog C. Van Der Velde G. (1988). “Structural aspects of aquatic plant communities,” in *Vegetation of Inland Waters*, ed. Symoens J. J. (Dordrecht: Springer), 113–153. 10.1007/978-94-009-3087-2_4

[B23] Elith J. Graham C. H. Anderson R. P. Dudik M. Ferrier S. Guisan A. (2006). Novel methods improve prediction of species’ distributions from occurrence data. *Ecography* 29 129–151. 10.1111/j.2006.0906-7590.04596.x

[B24] Ferreira M. T. Moreira I. S. (1995). “The invasive component of a river flora under the influence of Mediterranean agricultural systems,” in *Plant invasive General Aspects and Special Problems*, eds Pyšek P. Prack K. Rejmánek M. Wade M. (Amsterdam: SPB Academic Publishers), 117–127.

[B25] Ferreira M. T. Moreira I. S. (1999). River plants from an Iberian basin and environmental factors influencing their distribution. *Hydrobiologia* 415 101–107. 10.1023/A:1003837802366

[B26] Fielding A. H. Bell J. F. (1997). A review of methods for the assessment of prediction errors in conservation presence / absence models. *Environ. Conserv.* 24 38–49. 10.1017/S0376892997000088

[B27] Franklin J. (2013). Species distribution models in conservation biogeography: developments and challenges. *Divers. Distrib.* 19 1217–1223. 10.1111/ddi.12125

[B28] Franklin P. Dunbar M. Whitehead P. (2008). Flow controls on lowland river macrophytes: a review. *Sci. Total Environ.* 400 369–378. 10.1016/j.scitotenv.2008.06.018 18644618

[B29] Gasith A. Resh V. H. (1999). Streams in Mediterranean climate regions: abiotic influences and biotic responses to predictable seasonal events. *Annu. Rev. Ecol. Syst.* 30 51–81. 10.1146/annurev.ecolsys.30.1.51

[B30] Getsinger K. D. (1998). Chemical control research in the corps of engineers. *J. Aquat. Plant Manage.* 36 61–64.

[B31] Gómez C. M. Pérez-Blanco C. D. Batalla R. J. (2013). Tradeoffs in river restoration: Flushing flows vs. hydropower generation in the Lower Ebro River, Spain. *J. Hydrol.* 518 130–139. 10.1016/j.jhydrol.2013.08.029

[B32] Gozlan R. E. Mastrorillo S. Copp G. H. Lek S. (1999). Predicting the structure and diversity of young-of-the-year fish assemblages in large rivers. *Freshw. Biol.* 41 809–820. 10.1046/j.1365-2427.1999.00423.x

[B33] Gu R. Montgomery S. Austin T. A. (1998). Quantifying the effects of stream discharge on summer river temperature. *Hydrol. Sci.* 43 885–904. 10.1080/02626669809492185 29512009

[B34] Guillera-Arroita G. Lahoz-Monfort J. J. Elith J. Gordon A. Kujala H. Lentini P. E. (2015). Is my species distribution model fit for purpose? Matching data and models to applications. *Glob. Ecol. Biogeogr.* 24 276–292. 10.1111/geb.12268

[B35] Guisan A. Thuiller W. (2005). Predicting species distribution: offering more than simple habitat models. *Ecol. Lett.* 8 993–1009. 10.1111/j.1461-0248.2005.00792.x 34517687

[B36] Habib S. Yousuf A. R. (2014). Impact of mechanical deweeding on the phytophilous macroinvertebrate community of an eutrophic lake. *Environ. Sci. Pollut. Res.* 21 5653–5659. 10.1007/s11356-013-2470-7 24424481

[B37] Halstead J. M. Michaud J. Hallas-Burt S. Gibbs J. P. (2003). Hedonic analysis of effects of a nonnative invader (*Myriophyllum heterophyllum*) on New Hampshire (USA) Lakefront Properties. *Environ. Manage.* 32 391–398. 10.1007/s00267-003-3023-5 14753624

[B38] Hussner A. Stiers I. Verhofstad M. J. J. M. Bakker E. S. Grutters B. M. C. Haury J. (2017). Management and control methods of invasive alien freshwater aquatic plants: a review. *Aquat. Bot.* 136 112–137. 10.1016/j.aquabot.2016.08.002

[B39] Janauer G. A. Schmidt-Mumm U. Schmidt B. (2010). Aquatic macrophytes and water current velocity in the Danube River. *Ecol. Eng.* 36 1138–1145. 10.1016/j.ecoleng.2010.05.002

[B40] Jones J. I. Young J. O. Eaton J. W. Moss B. (2002). The influence of nutrient loading, dissolved inorganic carbon and higher trophic levels on the interaction between submerged plants and periphyton. *J. Ecol.* 90 12–24. 10.1046/j.0022-0477.2001.00620.x

[B41] Jowett I. G. Duncan M. J. (2012). Effectiveness of 1D and 2D hydraulic models for instream habitat analysis in a braided river. *Ecol. Eng.* 48 92–100. 10.1016/j.ecoleng.2011.06.036

[B42] Jowett I. G. Hayes J. W. Duncan M. J. (2008). *A Guide to Instream Habitat Survey Methods and Analysis.* Auckland: National Institute of Water & Atmospheric Research (NIWA).

[B43] Koch E. W. (2001). Beyond light: physical, geological, and geochemical parameters as possible submersed aquatic vegetation habitat requirements. *Estuaries* 24 1–17. 10.2307/1352808

[B44] Köhler J. Hachoł J. Hilt S. (2010). Regulation of submersed macrophyte biomass in a temperate lowland river: Interactions between shading by bank vegetation, epiphyton and water turbidity. *Aquat. Bot.* 92 129–136. 10.1016/j.aquabot.2009.10.018

[B45] Lahoz-Monfort J. J. Guillera-Arroita G. Wintle B. A. (2014). Imperfect detection impacts the performance of species distribution models. *Glob. Ecol. Biogeogr.* 23 504–515. 10.1111/geb.12138

[B46] Liaw A. Wiener M. (2002). Classification and Regression by randomForest. *R News* 2 18–22.

[B47] Liu C. Berry P. M. Dawson T. P. Pearson R. G. (2005). Selecting thresholds of occurrence in the prediction of species distributions. *Ecography* 28 385–393. 10.1111/j.0906-7590.2005.03957.x

[B48] Madsen J. D. (2000). Advantages and disadvantages of aquatic plant management techniques. *Lakeline* 20 22–34. 17453921

[B49] Madsen J. D. Chambers P. A. James W. F. Koch E. W. Westlake D. F. (2001). The interaction between water movement, sediment dynamics andsubmersed macrophytes. *Hydrobiologia* 444 71–84. 10.1023/A:1017520800568

[B50] Mainstone C. P. Parr W. (2002). Phosphorus in rivers - Ecology and management. *Sci. Total Environ.* 28 25–47. 10.1016/S0048-9697(01)00937-8 11846073

[B51] Mathur D. Bason W. H. Purdy E. J. Jr. Silver C. A. (1985). A critique of the in stream flow incremental methodology. *Can. J. Fish. Aquat. Sci.* 42 825–831. 10.1139/f85-105 23377706

[B52] Milner A. M. Brittain J. E. Castella E. Petts G. E. (2001). Trends of macroinvertebrate community structure in glacier-fed rivers in relation to environmental conditions: a synthesis. *Freshw. Biol.* 46 1833–1847. 10.1046/j.1365-2427.2001.00861.x

[B53] Moreira I. Ferreira T. Monteiro A. Catarino L. Vasconcelos T. (1999). Aquatic weeds their management in Portugal: insights and the international context. *Hydrobiologia* 415 229–234. 10.1023/A:1003847621640

[B54] Nikora V. Larned S. Nikora N. Debnath K. Cooper G. Reid M. (2008). Hydraulic resistance due to aquatic vegetation in small streams: field study. *J. Hydraul. Eng.* 134 1326–1332. 10.1061/(ASCE)0733-9429(2008)134:9(1326)

[B55] Noack M. Schneider M. Wieprecht S. (2013). “The habitat modelling system CASiMiR: a multivariate Fuzzy approach and its applications,” in *Ecohydraulics: An Integrated Approach*, eds Maddock I. Harby A. Kemp P. Wood P. J. (New York, NY: John Wiley & Sons), 75–91. 10.1002/9781118526576.ch4

[B56] O’Hare M. T. Baattrup-Pedersen A. Nijboer R. Szoszkiewicz K. Ferreira T. (2006). Macrophyte communities of European streams with altered physical habitat. *Hydrobiologia* 566 197–210. 10.1007/s10750-006-0095-2

[B57] Olden J. D. Jackson D. A. Peres-Neto P. R. (2002). Predictive models of fish species distributions: a note on proper validation and chance predictions. *Trans. Am. Fish. Soc.* 131 329–336. 10.1577/1548-8659(2002)131<0329:PMOFSD>2.0.CO;2

[B58] Perona P. Camporeale C. Perucca E. Savina M. Molnar P. Burlando P. (2009). Modelling river and riparian vegetation interactions and related importance for sustainable ecosystem management. *Aquat. Sci.* 71 266–278. 10.1007/s00027-009-9215-1

[B59] Peterson A. T. (1999). Conservatism of ecological niches in evolutionary time. *Science* 285 1265–1267. 10.1126/science.285.5431.1265 10455053

[B60] Pollen-Bankhead N. Thomas R. E. Gurnell A. M. Liffen T. Simon A. O’Hare M. T. (2011). Quantifying the potential for flow to remove the emergent aquatic macrophyte *Sparganium erectum* from the margins of low-energy rivers. *Ecol. Eng.* 37 1779–1788. 10.1016/j.ecoleng.2011.06.027

[B61] Pyšek P. Richardson D. M. Rejmánek M. Webster G. L. Williamson M. Kirschner J. (2004). Alien plants in checklists and floras: towards better communication between taxonomists and ecologists. *Taxon* 53 131–143. 10.2307/4135498

[B62] Raleigh R. F. Miller W. J. Nelson P. C. (1986). Habitat suitability index models and instream flow suitability curves: Chinook Salmon. *U.S. Fish Wildl. Serv. Biol. Rep.* 82:64.

[B63] Riis R. Biggs B. J. F. (2001). Distribution of macrophytes in New Zealand streams and lakes in relation to disturbance frequency and resource supply - a synthesis and conceptual model. *New Zeal. J. Mar. Freshw. Res.* 35 255–267. 10.1080/00288330.2001.9516996

[B64] Riis T. Biggs B. J. F. (2003). Hydrologic and hydraulic control of macrophyte establishment and performance in streams. *Limnol. Oceanogr.* 48 1488–1497. 10.4319/lo.2003.48.4.1488

[B65] Riis T. Suren A. M. Clausen B. Sand-Jensen K. (2008). Vegetation and flow regime in lowland streams. *Freshw. Biol.* 53 1531–1543. 10.1111/j.1365-2427.2008.01987.x 24587718

[B66] Schmolke A. Thorbek P. DeAngelis D. L. Grimm V. (2010). Ecological models supporting environmental decision making: A strategy for the future. *Trends Ecol. Evol.* 25 479–486. 10.1016/j.tree.2010.05.001 20605251

[B67] Schoelynck J. De Groote T. Bal K. Vandenbruwaene W. Meire P. Temmerman S. (2012). Self-organised patchiness and scale-dependent bio-geomorphic feedbacks in aquatic river vegetation. *Ecography* 35 760–768. 10.1111/j.1600-0587.2011.07177.x

[B68] Shields A. (1936). Anwendung der Aehnlichkeitsmechanik und der Turbulenzforschung auf die Geschiebebewegung. *Technology* 26 26.

[B69] Steffen K. Leuschner C. Mueller U. Wiegleb G. Becker T. (2014). Relationships between macrophyte vegetation and physical and chemical conditions in northwest German running waters. *Aquat. Bot.* 113 46–55. 10.1016/j.aquabot.2013.10.006

[B70] Steffler P. Blackburn J. (2002). *River2D Two-Dimensional Depth Averaged Model of River Hydrodynamics and Fish Habitat Introduction to Depth Averaged Modeling and User’s Manual.* Edmonton: University of Alberta.

[B71] Tena A. Ksiaszek L. Vericat D. Batalla R. J. (2013). Assessing the geomorphic effects of a flushing flow in a large regulated river. *River Res. Appl.* 29 876–890. 10.1002/rra.2572

[B72] VanDerWal J. Falconi L. Januchowski S. Shoo L. Storlie C. (2014). *SDMTools: Species Distribution Modelling Tools: Tools for Processing Data Associated with Species Distribution Modelling Exercises.* Available at: https://cran.r-project.org/package=SDMTools

[B73] Vereecken H. Baetens J. Viaene P. Mostaert F. Meire P. (2006). “Ecological management of aquatic plants: effects in lowland streams,” in *Macrophytes in Aquatic Ecosystems: From Biology to Management*, Caffrey J. M. Dutartre A. Haury J. Murphy K. J. Wade P. M. (Berlin: Springer), 205–210.

[B74] Wersal R. M. Madsen J. D. Gerard P. D. (2013). Survival of parrotfeather following simulated drawdown events. *J. Aquat. Plant Manage.* 51 22–26.

